# Do pet dogs reciprocate the receipt of food from familiar and unfamiliar conspecifics?

**DOI:** 10.1111/eth.13430

**Published:** 2024-01-10

**Authors:** Jim McGetrick, Leona Fux, Johannes Schullern-Schrattenhofen, Jean-Loup Rault, Friederike Range

**Affiliations:** 1 Domestication Lab, Konrad Lorenz Institute of Ethology, University of Veterinary Medicine, Vienna, Austria; 2 Department of Behavioural and Cognitive Biology, University of Vienna, Vienna, Austria; 3 Institute of Animal Welfare Science, University of Veterinary Medicine, Vienna, Vienna, Austria

**Keywords:** cooperation, dog, familiarity, mechanism, oxytocin, reciprocity

## Abstract

Reciprocity is one of the most prominent explanations for the evolution of stable cooperation. Although reciprocity has been studied for decades in numerous animal species and behavioural contexts, its underlying proximate mechanisms remain unclear. Domestic dogs provide a useful model species for the study of proximate mechanisms, though there are currently inconsistent findings regarding dogs’ propensity to reciprocate. Here, we investigated whether, after minimal training, pet dogs would press a button, which remotely controlled a food dispenser, to deliver food to an enclosure occupied by a helpful conspecific that had provided them with food or an unhelpful conspecific that had not provided them with food. We included an asocial control condition in which the enclosure was unoccupied and a social facilitation control in which the food delivery mechanism was non-functional. Whether subjects were familiar with the helpful and unhelpful conspecifics was also varied. In addition, to investigate potential mechanisms underlying reciprocity, we measured subjects salivary oxytocin concentration before and after they experienced the helpful and unhelpful acts. There was no effect of the previous helpfulness or the familiarity of the partner on the number of times subjects pressed the button. However, there was also no effect of the presence of a partner or the operationality of the food delivery mechanism on the number of button presses, indicating that subjects were not pressing the button to provision the partner. Moreover, the experience of the helpful or unhelpful act did not influence subjects’ salivary oxytocin concentration. Variation in findings of reciprocity across studies appears to correspond with differing training protocols. Subjects’ understanding of the task in the current study may have been constrained by the limited training received. Additional tests to verify subjects’ understanding of such tasks are warranted in future studies.

## 1 Introduction

Cooperation can be observed in many species in the animal kingdom and numerous behavioural contexts (Dugatkin, 1997; Taborsky et al., 2021). One of the most prominent explanations for the evolution of stable cooperation among unrelated individuals is reciprocity (or reciprocal altruism; Trivers, 1971). Reciprocity typically refers to the conditional exchange of resources or services with some delay in between such cooperative acts (but see Taborsky et al. [2021, 2016] for alternative definitions). Reciprocity occurs in nature among non-human animals. For example, primates have been shown to reciprocate the receipt of grooming, food and agonistic support (see Schweinfurth & Call, 2019b for a review) and vampire bats have been observed to donate blood meals to group members that have provisioned them on previous occasions (Carter & Wilkinson, 2013; Ripperger et al., 2019; Wilkinson, 1984). Similarly, in experimental settings, rats have been shown to provide food to conspecifics that have provided food to them (Rutte & Taborsky, 2008) and can use other commodities such as grooming to repay the receipt of food (Schweinfurth & Taborsky, 2018a). Despite decades of research on reciprocity and much theorizing, however, the proximate mechanisms, particularly cognitive and affective aspects, underlying such behaviour are not well understood (Brosnan & de Waal, 2002; Schino & Aureli, 2009, 2010, 2017; Schweinfurth & Call, 2019a).

Reciprocity has previously been considered cognitively demanding, requiring individual recognition, numerical discrimination, temporal discounting and memory of previous social encounters, leading to the suggestion that it should be rare if not absent in non-human animals (Stevens & Hauser, 2004). However, the current prevailing view is that advanced cognitive abilities may not be required for reciprocity, with simpler emotionally based mechanisms such as attitudinal reciprocity (de Waal, 2000) and emotional bookkeeping (Schino & Aureli, 2009, 2017) also giving rise to reciprocal interactions. According to the mechanisms of attitudinal reciprocity and emotional bookkeeping, the receipt of resources or services results in the recipient developing a positive attitude towards the donor, increasing the likelihood of the favour being reciprocated. Oxytocin, a neuropeptide hormone involved in social bonding and affiliation (Froemke & Young, 2021; Rigney et al., 2022), has been suggested to play a key role in emotionally mediated reciprocity (Freidin et al., 2017; Wittig et al., 2014), promoting bonding between individuals and, in turn, greater cooperation. Based on current evidence, such a mechanism is plausible. For example, grooming in chimpanzees has been shown to increase urinary oxytocin concentration in both the donor and the receiver (Crockford et al., 2013) while intranasal administration of oxytocin in vampire bats increased the amount of food donated and the amount of grooming donated by females to conspecifics (Carter & Wilkinson, 2015). Nevertheless, studies investigating the physiological underpinnings of reciprocity in non-human animals are scarce.

Domestic dogs are a potentially good model species to study the mechanisms underlying reciprocity in non-human animal species. Apart from pet dogs demonstrating prosocial behaviour, in the context of food giving (Dale et al., 2016; Quervel-Chaumette et al., 2015; but see Dale, Despraz, et al., 2019; Dale, Palma-Jacinto, et al., 2019) and rescue behaviour (Carballo et al., 2020; Van Bourg et al., 2020), military dogs have been shown to reciprocate the receipt of food from unfamiliar conspecifics in two studies (Gfrerer & Taborsky, 2017, 2018). In the first study, military dogs were more likely to pull a tray to deliver food to a conspecific in a neighbouring enclosure if they had previously received food from an unfamiliar conspecific themselves via the same apparatus, than if they had not received food from a conspecific (Gfrerer & Taborsky, 2017). In a subsequent study, Gfrerer and Taborsky (2018) demonstrated that military dogs would even provide food to a previously helpful unfamiliar conspecific using a different kind of action than that used by the helpful conspecific (i.e. pulling a tray vs. pressing a lever to open a box), exhibiting flexibility in their reciprocation and demonstrating that this behaviour is not simply based on copying of a partner’s actions.

Despite these seemingly robust findings of reciprocity in military dogs, a recent study on pet dogs failed to observe reciprocation of the receipt of food from humans. In a broadly similar experimental design to that used by Gfrerer and Taborsky (2017, 2018), helpful human partners provided food to a pet dog in a neighbouring enclosure by pressing a button that remotely controlled a food dispenser, while an unhelpful human refrained from pressing the button in separate sessions (McGetrick et al., 2021). The dog was later given the opportunity to provide food to the two different humans in return using the same action; although they pressed the button, they displayed no inclination to press the button for one partner type more than the other. Moreover, they also did not press the button more times if there was a human present in the neighbouring enclosure than if the enclosure was unoccupied or if the food delivery system was non-functional. These results indicate that the dogs’ button-pressing behaviour was not performed to provide a partner with food, and they also point towards a possible lack of understanding of the setup.

Apart from the fact that one study employed pet dogs whereas the others focused on military dogs, one potential reason for different outcomes between these studies could be the species of the partner. McGetrick et al. (2021) used humans as partners, whereas Gfrerer and Taborsky (2017, 2018) employed dogs. Dogs have been shown to treat humans and conspecifics differently in experimental prosociality studies. For example, dogs provided food to familiar conspecifics but not to humans by pulling a tray resulting in delivery of food to a neighbouring enclosure (Quervel-Chaumette et al., 2015; Quervel-Chaumette et al., 2016). Thus, in the current study, our first aim was to determine whether pet dogs would reciprocate the receipt of food from other pet dogs using a similar setup to that of McGetrick et al. (2021). However, given that familiarity has been shown to play a crucial role in governing pet dogs’ decision to donate food to a conspecific, with dogs donating to familiar but not unfamiliar conspecifics (Dale et al., 2016; Quervel-Chaumette et al., 2015), our second aim was to investigate the effect of familiarity on reciprocity. Our final aim was to determine whether changes in salivary oxytocin concentration were associated with the experience of a cooperative or non-cooperative act and predictive of later reciprocation, given oxytocin’s hypothesized role in mediating reciprocity.

Here, pet dogs experienced the receipt of food, via activation of a food dispenser, from a helpful conspecific, and on a separate occasion they experienced no receipt of food from an unhelpful conspecific who did not activate the dispenser. On the same day, they were given the opportunity to reciprocate with whichever partner type they experienced on that day. In a test condition, the subject could press a button to activate the food dispenser to provide the partner with food. In a social facilitation control, which controlled for the mere presence of a partner motivating the subject to act, the food dispenser was switched off and parts of the food delivery system were removed, rendering the food delivery mechanism non-functional. This allowed us to determine whether any button pressing observed was due to the subject wanting to provide food to the partner or whether the presence of the partner simply motivated the subject to act. This control was included as the presence of another individual has been shown to influence a subject’s behaviour, including in food provisioning studies (Dale et al., 2016; Jensen et al., 2006; Zajonc, 1965). In an asocial condition, no partner was present, to determine whether the subject pressed the button for non-social reasons.

## 2 Methods

### 2.1 Ethical approval

All procedures were approved by the animal ethics and animal welfare committee of the University of Veterinary Medicine, Vienna (protocol nos: ETK-82/05/2019; ETK-060/03/2020; and ETK 137/09/2020), in accordance with the Good Scientific Practices guidelines and national legislation. Additionally, dog owners were required to sign a consent form prior to participation in the study.

### 2.2 Subjects

In total, 72 pet dogs (*Canis familiaris*) participated in this study. Twenty-four pet dogs were included in the study as subjects (mean age ± SD: 5.63 ± 3.39 years; min. 1 year, max. 12 years). Twelve of these (6 f, 6 m) were tested with two conspecific partners with which they were familiar, and the other 12 (6 f, 6 m) were tested with conspecific partners with which they were unfamiliar. In addition to the 24 subjects, 2 unique partners were included in the study for each subject, to act as helpful and unhelpful partners (i.e. 48 partners in total; familiar, 15 f, 9 m; unfamiliar, 14 f, 10 m). None of these partners participated in the study as subjects. Thirty-three additional dogs that were initially recruited for the study did not take part either due to owner withdrawal or failure to pass the training criteria.

Participants were recruited via an online recruitment poster distributed on social media and through directly contacting owners who had participated in previous studies at the laboratory. Dogs were required to be at least 1 year of age and to be tolerant of conspecifics, generally. All subjects passed the training steps within a maximum of three sessions.

For the familiar subjects and partners, we attempted to balance the level of familiarity between the subject and the two partners and the degree to which the subject was related to each partner. Subjects either had to have lived with both partners or had to have had weekly interactions with both partners for at least 6 months prior to participation in the study. For 9 of the 12 subjects, the 2 partners were from the same household as the subject. For unfamiliar subjects and partners, the subject and partner had never met before participation in the study. Following recruitment, dogs were assigned to the role of subject, helpful partner, or unhelpful partner, depending on food motivation and the level of relatedness and familiarity with each other in the case of the familiar subjects and partners (i.e. we attempted to ensure that the level of familiarity and relatedness between the subject and both partners was equal). Dogs that were motivated to press the button were assigned the role of either subject or helpful partner and unmotivated dogs were assigned the role of unhelpful partner.

### 2.3 STRANGEness of study sample

Following recommendations made by the STRANGE framework (Webster & Rutz, 2020), we attempted to recruit a varied sample of pet dogs living in family homes using the approaches listed above. The sample with regards to subject age, sex and breed was relatively varied, though a large proportion were herding dogs (see Data S1). Previous experience with button-pressing tasks or food dispensers in the dogs’ home lives was not taken into account. Our assignment of roles (i.e. subject, helpful partner and unhelpful partner) based on initial motivation to press the button, and the different training experiences within the context of the study (see below), potentially introduced a bias such that the helpful and unhelpful partners may have differed behaviourally throughout the study.

### 2.4 General overview

This study was similar to experimental reciprocity studies carried out with dogs (Gfrerer & Taborsky, 2017, 2018; McGetrick et al., 2021) and rats (Rutte & Taborsky, 2007, 2008). Initially, the dogs went through a training phase to learn how to use the apparatus and/or to become habituated to the test setup. Subsequently, an experience phase and test phase took place. On a test day, the subject experienced either the helpful partner providing them with food, or the unhelpful partner refraining from providing food. On the same day, in the test phase, the subject was given the opportunity to provide food to this partner in a test condition. Two control conditions were also carried out – the social facilitation control condition and the asocial control condition. Thus, on each of the 2 test days, during the test phase, each subject had three experimental conditions: the test condition, the social facilitation control condition and the asocial control condition. The order in which the three experimental conditions (test condition, social facilitation control and asocial control) were carried out was randomized. On a second test day, the same procedure took place but with the other partner type (i.e. if the subject was tested with the helpful partner on the first day, they were tested with the unhelpful partner on the second test day). The order in which subjects experienced the two partner types was counterbalanced. In the current study, following McGetrick et al. (2021), the provision of food entailed pressing a button which resulted in dry dog food being released from a food dispenser. Saliva samples were obtained from the subjects before and after each experience phase session for oxytocin concentration analysis.

Prior to experience phase sessions with unfamiliar partners, the subject and the partner were brought to an outdoor dog enclosure to meet in a 10-min session in which the dogs were free to interact with each other. This was carried out to minimize the discomfort of seeing an unfamiliar dog in the test enclosure for the first time.

### 2.5 Setup

**Figure 1:**
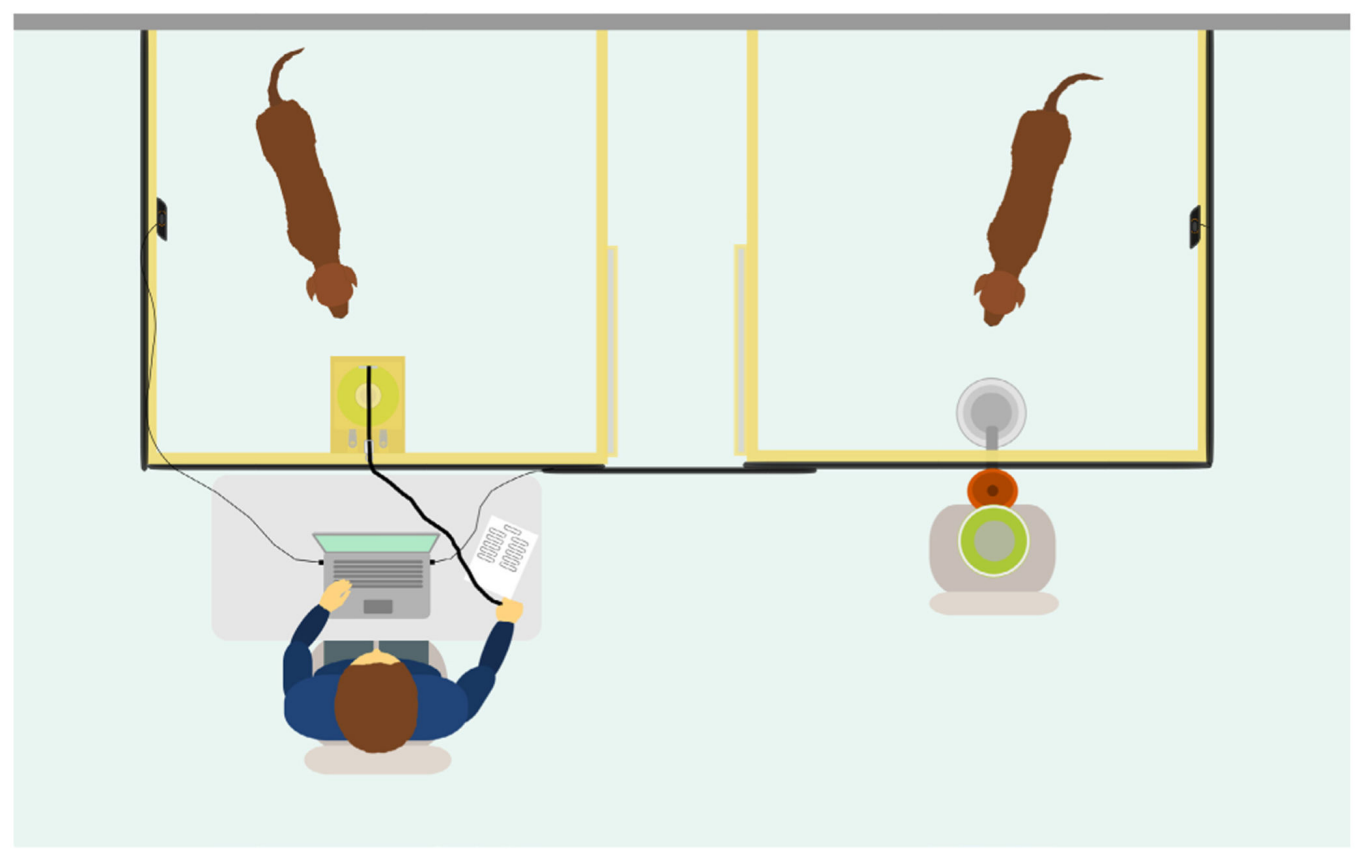
Experimental setup from above. The subject and partner were situated in two adjacent enclosures. The button inside the box was present in one enclosure while the food bowl into which food fell after activation of the food dispenser was present in the other. The experimenter sat outside the enclosure to the left and controlled opening of the box and monitored the two dogs via a webcam feed on their laptop.

The study was carried out primarily in a rectangular room (7m × 6 m) at the Clever Dog Lab of the University of Veterinary Medicine, Vienna. Most of the experiment took place within two adjacent square enclosures (1.5m × 1.5m) formed by wooden-framed, large-holed wire mesh fences (1.1 m height from ground) placed against a wall (see Figure 1). There was a space of 60 cm between the two enclosures to prevent dogs from having physical contact with each other when separated into the two enclosures. The outer fences were covered with black curtains to prevent visual distraction for dogs while they were inside the enclosures. The two inner fences facing each other were uncovered so that the dogs could see what was happening in the neighbouring enclosure. These two fences both had a sliding door consisting of a transparent Perspex sheet set within a wooden frame and allowed the dogs to be brought into each enclosure or to be quickly moved from one enclosure to the next.

Throughout the experience phase and test phase, the food dispenser (Trixie, Dog Activity Memory Trainer; cat no. 32040; TRIXIE Heimtierbedarf GmbH & Co. KG, Tarp, Germany) was positioned in front of the enclosure to the right (from the experimenter’s perspective when facing the enclosures), behind the black curtain, such that it was not visible to dogs inside the two enclosures. The dispenser was placed on a chair directly in front of a tube, which ran through the curtain and fence, such that activation of the dispenser resulted in food pieces falling down through the tube and into a bowl inside the enclosure. The dispenser was set up to release approximately five pieces of food when activated. The button that remotely controlled the food dispenser emitted an audible sound when pressed. It was set in a rubber green holder attached to a wooden base in the enclosure to the left, covered with a wooden box which was attached to the wooden base via hinges and springs on one side. A rope was attached to a handle on the wooden box and ran through a pulley wheel on the top of one of the fences. This rope allowed an experimenter to open the box from outside the enclosure by pulling; releasing the rope again resulted in the box closing due to the pressure from the springs. A trial was defined as a single presentation of the button with the button being available for 10 s, or until pressed and with an interval of 4 s in between each trial.

The experimenter sat at a desk in front of the enclosure to the left and could watch events in the two enclosures via a live webcam feed on their laptop. A chair was placed at either end of the room, close to the enclosures, for the owner(s) to sit on during the sessions. The owners were either visible to the dogs or not, depending on the phase of the study (see below). Two experimenters carried out the experimental work, each focusing on a random selection of approximately half of the subjects (for one of the subjects, one of the experimenters carried out the sessions with the helpful partner and the other carried out the sessions with the unhelpful partner).

### 2.6 Training

All stages of training took place in the same room as the experiment. Training was carried out to habituate the animals to the setup, to teach the subjects and helpful partners how to press the button and to teach the subjects the relationship between pressing the button and food being released into the neighbouring enclosure. Unhelpful partners were habituated to the test enclosures and general procedure but did not learn to press the button. The habituation and training steps for dogs in each role are outlined below. Training of the subjects lasted for a maximum of three sessions, each a maximum of approximately 45 min long. If a dog did not complete all the training steps in one session, the next session started one step back from where they ended in the previous session.

#### 2.6.1 Subjects

The habituation and training steps for subjects were divided into five stages.

##### Stage 1

In stage 1 of training, the button was placed in the middle of the experimental room in its green rubber holder, approximately 50 cm away from a food bowl (both the button and bowl were on the floor of the experimental room; the experimental enclosures were not used at this point though they were set up in the experimental room, and the food dispenser was not presented at this stage). The owner or the experimenter attempted to attract the dog’s attention towards the button either by using point or verbal cues, or by pressing the button or using a clicker. When the dog pressed the button, it emitted an audible sound and the experimenter placed approximately one to three pieces of dry food in the bowl. Once the dog had pressed the button and retrieved the food independently (i.e. without active encouragement, commands or ostensive cues from the owner or experimenter) five consecutive times, it proceeded to the next training stage. There was no strict time limit on pressing the button or retrieving the food at this stage.

##### Stage 2

Here, the button was placed inside the box in the experimental enclosure to the left. The box remained open (i.e. the button was always available to be pressed). The food dispenser was placed on a chair outside the same enclosure, in the same experimental room, with the tube passing through the fence, and the bowl was placed just below this inside the same enclosure, approximately .5 m away from the box with the button. Once the dog had pressed the button and retrieved the food 10 consecutive times independently, it proceeded to the next training stage. As with the previous stage, there was no strict restriction on the time available to press the button or to retrieve the food. Intermediate steps were required in most cases; this involved the owner or experimenter sitting in the enclosure with the dog or moving the button gradually into the box.

##### Stage 3

This stage was similar to the previous; however, opening and closing of the box (by pulling or releasing the rope) was introduced such that clearly defined trials were created. The button was available for the dogs to press for up to 10 s at a time (i.e. on each trial). Once the dog pressed the button within this period, the box was closed. The box remained closed for 4 s generally, but if the dog took longer than 4 s to retrieve the food, the experimenter did not open the box until the dog looked up. There was no limit on the time available for the dog to retrieve the food. If the dog was fearful of the movement of the box, the owner or experimenter sat with them and offered encouragement or opened and closed the box by hand initially.

During this stage, the sliding door of the dog’s enclosure was closed and all outer fences and the opening to the corridor between the two enclosures were covered with black curtains, preventing the dog from seeing their owner or the experimenter. The dogs were required to press the button and retrieve the food independently on 10 consecutive trials before proceeding to the next training stage. The food bowl was still present in the same enclosure as the button and the dispenser was still just outside this enclosure.

##### Stage 4

Here, the food bowl, tube and dispenser were moved to the second enclosure and the sliding doors between the two enclosures were left open. This meant that after pressing the button, food was released into the bowl in the neighbouring enclosure and the dog had to move into the neighbouring enclosure to retrieve it. The dogs were required to succeed (i.e. press the button and retrieve the food) on 10 consecutive trials independently before moving to the next stage of training. As with the previous stage, the button was available for the dogs to press for up to 10 s at a time. Once the dog pressed the button within this period, the box was closed. There was no limit on the time available for the dog to retrieve the food. The box remained closed for 4 s (or longer if it took longer to retrieve the food). Motivational trials, which were carried out later, resembled this stage of training.

##### Stage 5

Here, the setup was the same as that in stage 4 but the sliding doors between the enclosures were closed. Thus, if the dog pressed the button, food would be released into the bowl in the neighbouring enclosure but, despite being able to see it, the dog would not be able to reach it. Ten trials of this stage were carried out. The dog was allowed to press the button up to a maximum of 10 times to proceed to the experiment, although it was not required to press 10 times, as this stage of training was included only to give them the opportunity to see that if the sliding doors were closed and the button was pressed, the food would be released into the bowl but they would not be able to reach it. Following this stage, the dogs were given five more trials resembling stage 4, to maintain their motivation to press the button.

#### 2.6.2 Helpful partners

Training of the helpful partners was identical to that of the subjects, with the addition of two final training stages. The first of these additional training stages was identical to stage 5 of training; however, the experimenter rewarded them with dry food treats irregularly until they reached a performance of five successful trials in a row, without reward. Finally, the dogs were exposed to an identical training stage but with a second dog in the neighbouring enclosure, eating the food. These training stages were carried out to increase the likelihood of the helpful partners performing reliably in the experience phase of the study.

#### 2.6.3 Unhelpful partners

The unhelpful partner was not trained to press the button but was habituated to aspects of the experimental situation. In a first stage, the dog was guided into the enclosure with the box and the button. The box was opened by the experimenter, using the rope, for 10 s and closed for 4 s until the dog no longer seemed scared of openings of the box. If the dog was fearful of the movement of the box, the owner or experimenter sat with it and opened and closed the box directly by hand. If the dog showed interest in the button, it was not rewarded.

The second stage aimed to habituate the unhelpful partner to the sound of the dispenser and food falling into the bowl. The dog was guided into the neighbouring enclosure. The experimenter activated the food dispenser by hand resulting in food falling through the tube into the bowl. This act was carried out until the dog displayed no discomfort upon hearing the dispenser or the food falling into the bowl. When the dog seemed comfortable, it was deemed ready to participate as an unhelpful partner.

### 2.7 Experience phase

#### 2.7.1 General procedure

In the experience phase, the subject experienced the receipt of food from the helpful partner in the neighbouring enclosure on a test day and experienced receiving no food from the unhelpful partner, who had access to the button in the neighbouring enclosure, on another day. Generally, at the beginning of each session, the subject was brought into the enclosure to the right with the food bowl and the partner was then brought into the enclosure with the button inside the box. The experience phase lasted for 10 trials (i.e. 10 presentations of the button to the partner). The button was presented for 10 s unless the partner pressed the button, in which case the box was closed again. The box remained closed for 4 s in between each presentation. Helpful partners pressed the button due to their training, whereas the unhelpful partners never pressed the button. The helpful partner did not always press the button on every trial (mean presses ± SD: 8.79 ± 1.53; median: 9; min. 4, max. 10). After the first five trials, there was a 2-min break before beginning the final five trials. During the 2-min break, the subject was typically brought out of the test room while the partner remained in the test room. During the 2-min break, the helpful partner was rewarded for having pressed the button and was also given some motivational sessions to help maintain the button-pressing behaviour while the subject waited outside. In cases in which the subject and partner came from the same household, the owner could only handle one dog at a time and typically left the room with the subject during this break. However, some unhelpful partners were uncomfortable being left in the room alone with the experimenter. In these cases, the owner brought the unhelpful partner out of the room and the subject, who seemed more comfortable being in the room, remained there with the experimenter.

Following the last trial of the experience phase, the partner was escorted out of their enclosure and out of the experimental room. The subject was also let out of their enclosure but remained in the experimental room for several reasons. First, saliva sampling was carried out with the subject (see below). Second, a motivation session was carried out with the subject prior to the test phase; therefore, the partner was not needed at this point. Third, after experience phase sessions with the unhelpful partner, the subject was given approximately the amount of food it would have received had it been a session with a helpful partner. This was to control for the effect of having received food, or satiety, on the subject’s behaviour later in the experiment and on salivary oxytocin measurements. After experience phase sessions with the helpful partner, the subject was given five pieces so that the subjects’ experience on the 2 days outside the sessions with the two partner types was as similar as possible. In both cases, the experimenter used the same dry food as was used in the food dispenser throughout the study. The experimenter provided the food to the subject by placing the pieces of food in a separate food bowl outside the experimental enclosures but inside the experimental room. This procedure was carried out outside the experimental enclosures to reduce the likelihood of this food receipt being associated with the action or inaction of the partners.

During breaks, the subject and partner did not come into physical contact with each other. With the exception of the 2-min break in the middle of the experience phase session, the partner was removed from the test enclosure and test room before the subject was released from its enclosure, and the partner remained outside the room until the subject was back in its enclosure.

#### 2.7.2 Saliva sampling

One saliva sample was collected by the experimenter immediately before the experience phase sessions and a second sample was taken approximately 10 min later (approximately 4 min after the last button press). No salivation stimulant was used. Saliva was collected using Salimetrics Children’s Swabs (SalivaBio). Swabs (12.5 cm long × .8 cm diameter) were cut into four pieces of approximately equal length. With a gloved hand, the experimenter gently inserted a swab into the dog’s lower cheek on each side. The swab was left in the dog’s mouth for approximately 1–2 min. If a dog rejected the swab, the experimenter reintroduced it and aimed to collect the sample within 1–2 min to avoid changes in oxytocin concentration due to the sampling procedure. The sample was then withdrawn, put back in a plastic tube case and stored in a −20°C freezer. Saliva samples were thawed on ice and centrifuged for 20 min at 4°C at 1500 *g*, and carefully avoiding food particles if present in the sample, the supernatant was transferred to a tube kept at −20°C until being assayed.

### 2.8 Test phase

The test phase with each partner took place after the experience phase session with that partner, on the same day, and consisted of three experimental conditions (see below). In each condition, the subject was brought into the enclosure to the left with the button before the partner was brought into the room and directed into the enclosure to the right with the food bowl. Each condition lasted for 20 trials (i.e. 20 presentations of the button) in which the subject was free to press the button. The box remained open on each trial for 10 s, unless the subject pressed the button, in which case it was closed immediately. The box remained closed for 4 s in between each trial. Before each condition, and at the end of the test phase, five motivational sessions were carried out with the subject to maintain their interest in pressing the button. These sessions matched stage 4 of the training. A 1-min break separated the motivational trials from the test conditions. The partner remained outside the room while these motivational sessions took place.

#### 2.8.1 Conditions

##### Test condition

In the test condition, if the subject pressed the button, food would be released from the dispenser and would fall through the tube into the food bowl for the partner to consume.

##### Social facilitation control condition

In the social facilitation control, the food bowl and tube were removed from the partner’s enclosure and the food dispenser was switched off. This meant that pressing the button did not result in food being released into the partner’s enclosure.

##### Asocial control condition

The asocial control condition was similar in terms of setup to the test condition. However, no partner was present in the enclosure with the food bowl.

### 2.9 Analyses

#### 2.9.1 Behaviour coding

Coding was carried out with the software Loopy (loopbio GmbH, Vienna, Austria; http://loopbio.com/loopy/) using the footage from the webcams. The number of times the subject pressed the button in each condition of the test phase, and the number of times the helpful partner pressed the button in the experience phase, were coded. If the subject attempted to press the button but failed to activate the dispenser (e.g. if it only touched the green rubber holder), this was included as a button press. Only one button press was counted per trial. One experimenter coded all videos with the familiar partners and another experimenter coded all videos with the unfamiliar partners. Both experimenters coded the same 20% of all videos for interobserver reliability analysis.

#### 2.9.2 Salivary oxytocin analysis

Analyses to determine oxytocin concentration of the saliva samples were conducted by ELISA using the Cayman Chemical Oxytocin ELISA kit (cat no. 500440; Cayman Chemical, Ann Arbor, MI, USA), running each sample in duplicate. Saliva samples were analysed without performing a solid-phase extraction as it is not required for dog saliva (MacLean et al., 2018). Intra-assay CVs were <7.1%. Samples from the same dog were analysed on the same plate to minimize the influence of interassay variation according to the within-subject test design.

#### 2.9.3 Statistical analyses

All models were fitted in R (versions 4.2.2–4.3.0; R Core Team, 2023). The packages and functions used in each case are given below. Model diagnostics were carried out and model stability was assessed using functions kindly provided by Roger Mundry.

##### Number of times the subject pressed the button

To determine whether there was an effect of condition, for each partner type, in terms of both helpfulness and familiarity, on the number of times the subject pressed the button, we fitted a Generalized Linear Mixed Model (GLMM) with a binomial error distribution and a logit link function (McCullagh & Nelder, 1989). To enter the response variable, we used the ‘cbind’ function including the number of times the subject pressed the button and the number of times that they did not press the button, out of 20. In the model we included condition, partner helpfulness and partner familiarity, with a three-way interaction. These were the main terms of interest. To control for their potential effects, as fixed effects we included condition order (i.e. the order in which the conditions were carried out; 1, 2 and 3), partner helpfulness order (i.e. the order in which the subject experienced the helpful and unhelpful partners; 1 and 2), subject age, subject sex, partner age, partner sex and experimenter ID. Age, condition order, partner helpfulness order and partner age were z-transformed to a mean of 0 and a standard deviation of 1 prior to inclusion in the model to allow for easier interpretation of results and to ease model convergence.

We included the random intercept effect of subject and observation (i.e. the identity of each individual session; hereafter ‘observation level random effect’). The observation-level random effect was included to account for session-to-session variation in the propensity to press the button. In addition to random intercepts, to avoid overconfidence regarding the precision of estimates for the fixed effects, and to ensure that the type *I* error rate remained at the nominal level of 5%, we included all theoretically identifiable random slopes (Barr et al., 2013; Schielzeth & Forstmeier, 2009). Condition, partner helpfulness, condition order, partner helpfulness order, partner sex and partner age were all included as random slopes within the random effect of subject. Condition, partner helpfulness and partner sex were manually dummy coded and centred prior to inclusion as random slopes.

The model was fitted using the function ‘glmer’ from the package ‘lme4’ (version 1.1-33; Bates et al., 2015). The model could only be fitted once the correlations between the random slopes and the random intercept of subject were removed. The model was not overdispersed (dispersion parameter: .551). We assessed collinearity by determining variance inflation factors (VIFs; Field, 2005) using the function ‘vif’ from the package ‘car’ (3.1-2; Fox & Weisberg, 2019). This was applied to a linear model with the number of presses as the response variable and with no interactions or random effects. Collinearity did not appear to be an issue (maximum VIF: 1.28).

To assess model stability at the level of the estimated coefficients and standard deviations, we excluded the levels of the random effects one at a time (Nieuwenhuis et al., 2012). This revealed the model to be of good stability, generally.

To test of the effects of condition, partner helpfulness and partner familiarity and their interactions, we conducted a full-null model comparison to avoid cryptic multiple testing (Forstmeier & Schielzeth, 2011). The null model lacked these terms but was otherwise identical to the full model. The full-null model comparison was based on a likelihood ratio test (Dobson, 2002) using the R function ‘anova’ and setting the ‘test’ argument to ‘Chisq’. The sample for this model included a total of 144 observations across 24 subjects.

To assess interobserver reliability, we calculated the intraclass correlation coefficient using the function ‘icc’ in the package ‘irr’ (version 0.84.1; Gamer et al., 2019), setting the ‘model’ argument to ‘twoway’ and the ‘type’ argument to ‘consistency’. Interobserver reliability was excellent (ICC = .988, *n*_observations_ = 30, *n*_raters_ = 2, *p* < .001).

##### Salivary oxytocin concentration

To determine whether there was an effect of the experience of the helpful or unhelpful partner for each level of familiarity on salivary oxytocin concentration, we fitted a Linear Mixed Model (LMM). The response variable was log-transformed prior to inclusion in the model. The original response was in pg/mL. The structure of the remainder of the model was the same as that above with exceptions highlighted here. Condition and condition order were not present in the model as they were not applicable to the experience phase. A three-way interaction was included among time point (before and after), partner helpfulness and partner familiarity. These terms and their interactions were the main terms of interest. Subject was the only random intercept effect included in the model and no random slopes were included.

The model was fitted using the function ‘lmer’ from the package ‘lme4’ (version 1.1-33; Bates et al., 2015). Diagnostic plots were used to assess normality of the residuals and heteroscedasticity and no issues were detected. Collinearity was also not an issue (maximum VIF: 1.13). Model stability was excellent.

To test of the effects of time point, partner helpfulness and partner familiarity and their interactions, we conducted a full-null model comparison, as above. The null model lacked these terms but was otherwise identical to the full model. The sample for this model included a total of 71 observations across 22 subjects.

## 3 Results

### 3.1 Number of times the subject pressed the button

**Figure 2:**
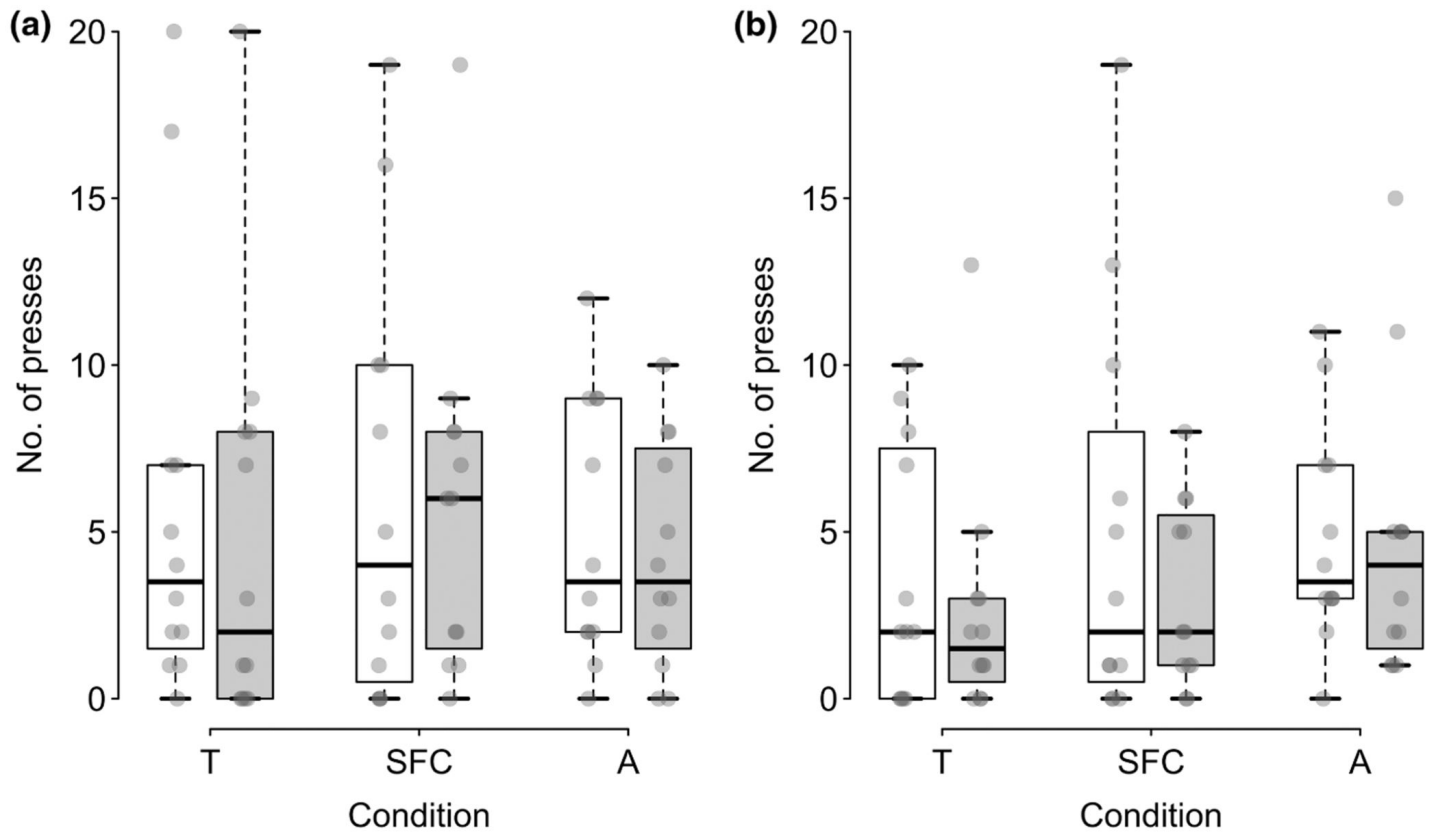
Number of times the subjects pressed the button for familiar partners (a) and unfamiliar partners (b) in each condition (T, test; SFC, social facilitation control; AC, asocial control) of the test phase with helpful (white) and unhelpful (grey) partners. Boxes display the interquartile range, black horizontal bars represent the median, whiskers represent the range of data points within 1.5 times the interquartile range from the upper and lower hinge and transparent grey points represent individual data points.

There was no effect of condition, partner type, partner familiarity or their interaction on the number of times the subjects pressed the button (full-null model comparison: *χ*^2^ = 10.979, *df* = 11, *p* = .445; see Figure 2).

#### 3.1.1 Salivary oxytocin concentration

**Figure 3:**
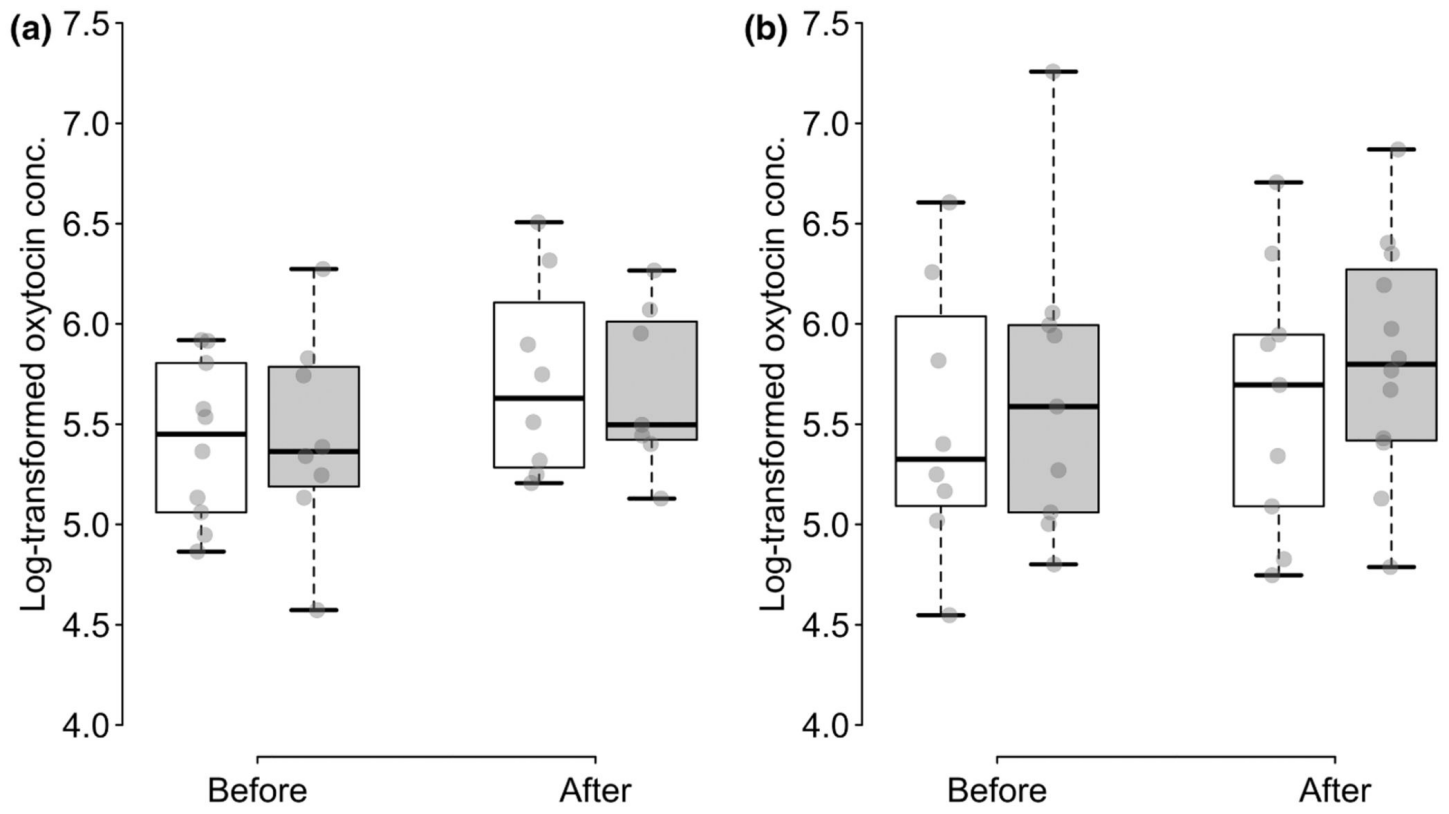
Log-transformed salivary oxytocin concentration before and after the experience phase with familiar (a) and unfamiliar (b) partners and with helpful (white) and unhelpful (grey) partners. Boxes display the interquartile range, black horizontal bars represent the median, whiskers represent the range of data points within 1.5 times the interquartile range from the upper and lower hinge and transparent grey points represent individual data points.

There was no effect of time point, partner helpfulness, partner familiarity or their interaction on salivary oxytocin concentration (full-null model comparison: *χ*^2^ = 6.686, *df* = 7, *p* = .462; see Figure 3).

## 4 Discussion

In the current study, subjects did not press the button more times for the helpful partner than for the unhelpful partner regardless of whether the partner was familiar to them or not. There was also no difference in the number of times subjects pressed the button in the control conditions compared with the test conditions, indicating that button pressing was not carried out to provide the partner with food and that subjects may not have fully grasped the task. Moreover, salivary oxytocin concentration did not change after experiencing helpful or unhelpful acts.

Although these findings corroborate those of a previous study investigating dogs’ reciprocation of food receipt from humans (McGetrick et al., 2021), they conflict with two studies on reciprocity among military dogs (Gfrerer & Taborsky, 2017, 2018). We did not set out to replicate Gfrerer and Taborsky’s (2017, 2018) studies directly. One of our initial aims was to determine whether McGetrick et al. (2021) failed to observe reciprocity due to the experimental setup or due to the partner species being human. As a result, our study design and selection of subjects differed from Gfrerer and Taborsky’s (2017, 2018) studies. Nevertheless, it is worth questioning why they observed reciprocity whereas we did not.

A failure to comprehend the experimental setup could prevent study participants from demonstrating a particular trait (see Brosnan, 2018 for a discussion). It is conceivable that our subjects did not understand the setup sufficiently whereas Gfrerer and Taborsky’s (2017, 2018) subjects did. One difference of note is that Gfrerer and Taborsky’s (2018) subjects did not provide food to an empty enclosure whereas ours did. A relatively high baseline performance (i.e. pressing the button in the asocial condition) does not necessarily indicate a lack of understanding of the task nor does it preclude differential responses based on partner type: dogs in the prosociality studies of Quervel-Chaumette et al. (2015) and Dale et al. (2016) both provided food to an empty enclosure but provided food significantly more times when a familiar conspecific occupied the enclosure. Nonetheless, the provision of food to an unoccupied enclosure does cast doubt over the subjects’ understanding and differences in the subjects’ understanding of the setup could explain the conflicting results between the reciprocity studies.

There are a couple of possible explanations for why our subjects might not have understood the setup, in contrast with the subjects of Gfrerer and Taborsky (2017, 2018). One major difference between our study and those with the military dogs, which could have influenced understanding, is the training protocol. Our training was limited to a maximum of three training sessions, each lasting approximately 45 min. This contrasts starkly with the military dog studies which had 14–19 days of training, each with two sessions per day. As part of our training, the subjects learned how to activate the food dispenser to release food and they experienced the impact of the doors between the enclosures being closed on their ability to reach the food. However, unlike the military dog studies (and successful reciprocity studies with rats; Dolivo & Taborsky, 2015; Rutte & Taborsky, 2007; Schweinfurth & Taborsky, 2016, 2018b), they did not experience exchanging roles with a partner and they were not specifically trained to press the button reliably. We opted for minimal training to strike a balance between the subjects grasping the mechanism, while not training a specific behavioural outcome. It is still possible, however, that our training was insufficient to allow the subjects to understand the relationship between button pressing and food delivery.

In this context, the food delivery system used in our study is also worth highlighting. It is possible that the non-mechanical connection between the button and the food dispenser caused problems for the dogs. The subjects quickly learned how to activate the dispenser, but it is unclear what they understood about the connection or how they perceived the system. Our social facilitation control in which some features of the food delivery system were missing may have been particularly difficult to understand (though the difference between this and test condition should have been stark given the absence of salient cues such as the sound of the food dropping into the bowl and the sound of the partner feeding). Although means–end understanding in dogs does not appear strong, at least in string-pulling tasks (Osthaus et al., 2005; Range et al., 2012), with clear mechanical connections, like in tray-pulling paradigms, there are more visual cues that can inform the participants about the relationship between their own or their partner’s action and the outcome. For example, if the partner is pulling a tray to draw food towards the subject, its movements are synchronized with the movements of the tray and food, and there is a clear connection between the tray and the partner. It is important to acknowledge, however, that Dale et al. (2016) observed prosociality in dogs employing a token choice task which, similarly, did not exploit a clear mechanical connection (see also Bräuer et al., 2013). Nevertheless, even if the lack of a mechanical connection is not an issue in terms of subjects understanding the consequences of their own action, it may create difficulties in understanding the significance of the act of a helpful partner. The inability to link the partner’s actions to food release while being able to link one’s own actions to food release would explain why non-mechanical connections could be used in prosociality but not reciprocity studies.

Pet dogs certainly can attend to the button-pressing action of a human partner, including when this results in the release of food via a non-mechanical mechanism. Recently, pet dogs were shown to be capable of coordinating their own button pressing with that of a human partner to obtain food treats using a similar non-mechanical connection to ours (Martínez et al., 2023). This finding appears to contradict the notion that dogs in our study could not grasp the significance of the partner’s behaviour. However, the context in which the action in Martínez et al.’s (2023) study took place, as well as the training protocol, differed substantially from ours. Of particular note, those subjects were trained over multiple sessions with most subjects receiving over 20 blocks of 20 trials each, before entering the main test. Moreover, the partners in Martínez et al.’s (2023) study were human unlike in our study in which the partners were dogs; it is plausible that the likelihood of attending to the actions of a human compared with a conspecific differ in such settings. Although these results demonstrate that dogs can pay attention to the button-pressing action of a human partner in a food-related context, it is not clear if the dogs in our study were similarly attentive.

Aside from reciprocity, it is surprising that the subjects did not display any prosociality in this study. The test sessions conducted here were almost identical to those of food-based prosociality studies with dogs (Dale et al., 2016; Quervel-Chaumette et al., 2015, 2016). In this regard, our test phase can be viewed in isolation as a prosociality study. Following Quervel-Chaumette et al. (2015) and Dale et al. (2016), one would have expected to observe food donation in the test condition above levels in the controls, at least when partners were familiar. The fact that this did not occur suggests some fundamental difference exists not only between our study and those of Gfrerer and Taborsky (2017, 2018) but also between our study and the aforementioned prosociality studies. All four of these studies employed more extensive training procedures than ours. Perhaps future investigations could titrate the training procedure to determine the adequate amount of training required for prosociality or reciprocity to emerge and ultimately determine what crucial aspects individuals must learn in this process. Moreover, given that all four studies above employed a different food delivery mechanism than us, future studies could also investigate the influence of the understanding of the food delivery mechanism.

Apart from training differences, it is also important to keep in mind that the samples used our reciprocity studies differed demographically from the military dog studies. Gfrerer and Taborsky’s (2017, 2018) subjects were all military dogs, whereas ours were pet dogs living in family homes. The training and selection history of military dogs outside the study context could facilitate a better performance within the study. Inhibitory control has, for example, been shown to be an important trait for explosive detection in police dogs (Tiira et al., 2020) and inhibitory control has been suggested to be important for reciprocity (Stevens & Hauser, 2004; though it may not be involved in emotionally mediated reciprocity; Schino & Aureli, 2017; Schweinfurth & Call, 2019a); it is plausible that military dogs have been selected for such traits and also that they develop these throughout their training. Furthermore, Gfrerer and Taborsky’s (2017, 2018) samples were homogenous in terms of breed and background in contrast with our samples which comprised a variety of pet dogs living in a human household. It is conceivable that such similarity among Gfrerer and Taborsky’s (2017, 2018) dogs facilitated attention to important social cues in the experimental context.

Further to variation in training, study samples and the food delivery paradigm, the actual action required of subjects in the different studies deserves mentioning. One of the successful prosociality studies (Quervel-Chaumette et al., 2015) and two of the successful reciprocity studies (Gfrerer & Taborsky, 2017, 2018) with dogs employed a tray-pulling paradigm which required the dogs to pull a tray with their mouth in order to deliver food. The second successful prosociality task with pet dogs involving food provisioning required the dogs to touch a token with their nose. The action performed in our study was pressing a button with a paw which differs considerably from pulling an object with the mouth or touching an object with the nose. It seems unlikely, however, that the exact action was an important factor in determining the outcome of the study, as one of the actions used by Gfrerer and Taborsky (2018), successfully demonstrating reciprocity in dogs, was pressing a lever with the paw to open a box, which is quite similar to pressing a button. Moreover, paradigms in which dogs have to press a button to obtain food have been successfully implemented in at least two other studies with pet dogs in contexts requiring them to pay attention to partner behaviour (Essler et al., 2017; Martínez et al., 2023), though the context was quite different.

It is important to acknowledge that apart from the cooperative (or non-cooperative) behaviour of the partner being unnatural, it was also a sham. The helpful partners were simply trained to press the button and the unhelpful partners were not trained to press the button at all, following the approach taken in reciprocity studies with dogs and rats. The helpful and unhelpful partners most likely had no greater understanding of the situation than the subjects. Consequently, they would not have exhibited any affective expressions or communication that might be associated with natural cooperation (Massen et al., 2019) and that might be important for the receiver to understand the partner’s behaviour as cooperative or non-cooperative. The importance of such features in reciprocity studies is currently unclear. In humans, it has been shown that the perception of the intentions of others is important in cooperative decision making (McCabe et al., 2003). However, Schweinfurth (2021) showed that only the outcome (i.e. receiving food or not receiving food) rather the willingness or ability of the partner to provide food influences rats’ decision to reciprocate. We do not know, nonetheless, whether the intentionality or unintentionality of the cooperative act was evident to subjects in our study or in the previous reciprocity studies with dogs (Gfrerer & Taborsky, 2017, 2018; McGetrick et al., 2021). It is particularly noteworthy, however, that in the context of food giving by humans, dogs have been shown to distinguish between similar actions that have different underlying intentions, regardless of the outcome (Völter et al., 2023).

In future experimental studies on reciprocity, it may be beneficial to induce naturally occurring cooperative behaviours (see Schweinfurth et al., 2017, and Schweinfurth & Taborsky, 2018a, for an example). It seems likely that naturally occurring cooperative behaviours are replete with a physical and communicative richness that cannot easily be replicated in a setting like ours. It is worth highlighting, however, that although artificial setups are often questionable in animal cognition studies due to ecological validity, with domestic and especially pet dogs, artificial setups are arguably more ecologically valid given that dogs are used to interacting with man-made objects such as food dispensers on a daily basis and have evolved to live around humans (Coppinger & Coppinger, 2016; Marshall-Pescini et al., 2017; Perri et al., 2020). Nevertheless, we would expect natural cooperative behaviours to be more salient.

To improve the generalizability of our findings, we recruited a relatively varied sample of pet dogs, with regards to age, sex and breed, from family homes, though many of the participants were herding breeds. Despite our attempt to recruit a representative sample of pet dogs, as with most behaviour and cognition studies with pet dogs, the sample was undoubtedly biased towards owners who are motivated to carry out activities with their dogs, as well as breeds and individual dogs that are easier to train. Moreover, given that the roles of helpful partner and unhelpful partner were assigned based on initial motivation to press the button, and the subsequent training or habituation differed for these dogs, it is possible that, apart from the button pressing behaviour, the two partners differed in their general behaviour throughout the study, thereby biasing the subjects’ response.

One important feature of the current study worth noting is that two unique partners were used for each subject. This allowed us to avoid pseudo-replication, a feature common in such setups due the limited number of individuals typically available in captive settings, and due to the often demanding training protocols for partners. Such variation in partner identity could be incorporated into future studies focusing on dyadic interactions to produce more robust results.

Regarding the salivary oxytocin concentration, the lack of significant changes in the experience phase is not particularly surprising. If changes in oxytocin concentration were associated with reciprocity, one would not necessarily expect to observe such effects in a study in which reciprocity was not observed. However, future studies could incorporate a positive control for the measurement of salivary oxytocin concentration in dogs. For example, one could include a condition in which the owner or trainer interacts positively with the dog, including stroking, as such affiliative interactions have been shown to result in increases in dogs’ salivary oxytocin concentration (López-Arjona et al., 2021; MacLean et al., 2017; Ogi et al., 2020).

In conclusion, we did not find evidence for reciprocation of the receipt of food regardless of whether dogs were paired with familiar or with unfamiliar cooperative and uncooperative conspecifics, though it is not clear whether the subjects fully understood the task. Moreover, no change in salivary oxytocin concentration was observed after the experience of the cooperative or uncooperative act of the partner. Our results contrast with those that previously showed reciprocity and prosociality in domestic dogs. Differences in training procedures are the most plausible explanation for such variation in findings. In future studies, it would be worthwhile to determine the amount of training required for reciprocity or prosociality to emerge and also to include additional steps to verify that the subjects understand the setup. Insights could also be gained into the cognitive mechanisms underlying reciprocity and prosociality if the important features that participants learn in this process are identified.

## Supplementary Material

Supplementary Materials

## Data Availability

The data used in the analysis for this manuscript are included in the Supporting Information.
